# Classifying Motorcyclist Behaviour with XGBoost Based on IMU Data

**DOI:** 10.3390/s24031042

**Published:** 2024-02-05

**Authors:** Gerhard Navratil, Ioannis Giannopoulos

**Affiliations:** Department for Geodesy and Geoinformation, TU Wien, Wiedner Hauptstr. 8-10, 1040 Vienna, Austria; igiannopoulos@geo.tuwien.ac.at

**Keywords:** behaviour classification, XGBoost, motorcycle, navigation, traffic, IMU, experiment

## Abstract

Human behaviour detection is relevant in many fields. During navigational tasks it is an indicator for environmental conditions. Therefore, monitoring people while they move along the street network provides insights on the environment. This is especially true for motorcyclists, who have to observe aspects such as road surface conditions or traffic very careful. We thus performed an experiment to check whether IMU data is sufficient to classify motorcyclist behaviour as a data source for later spatial and temporal analysis. The classification was done using XGBoost and proved successful for four out of originally five different types of behaviour. A classification accuracy of approximately 80% was achieved. Only overtake manoeuvrers were not identified reliably.

## 1. Introduction

Human behaviour recognition has been a research topic for decades. Behaviour recognition requires a method to monitor the person of interest. This is often done by observation, either directly by observers or by using cameras [1,2]. However, other sensors have been adopted as well, e.g., Global Navigation Satellite Systems (GNSS) [3] or inertial sensors [4,5,6]. The use of videos provides a lot of information but requires cameras covering the whole area of interest. Such infrastructure can be installed inside a building or even covering (at least parts of) a city, but this is challenging for an entire country. Additionally, there is a potential privacy problems with using videos [7]. Cameras also have an advantage. The monitored person does not need to carry devices, e.g., a smartphone, because cameras are already installed. Khan et al. [7] attempted to overcome the problems of videos by using the interaction between human bodies and signals from WiFi networks to detect activities. In general, a large variety of sensors can be used to monitor human behaviour, but the optimal choice in terms of efficiency, legality, and economy depends on the application scenario. However, there is also a large variety of algorithms that can be applied. Fayad et al. [8], for example, published an extensive lists of Kinect-based fall detection approaches. Eye-trackers have also been investigated to monitor human behaviour, e.g., in the context of personnel selection for pilots and air traffic controllers [9]. However, there are only a few approaches that do not rely on dense infrastructure or sensors carried by the monitored person or restricting the geographic area of observation.

The geographic area of observation is essential when dealing with human behaviour recognition in the field of navigation. Navigation is a standard problem in everyday life and thus there is a vast amount of scientific literature on the topic. Questions range from geometrical modelling of road networks to computing optimal paths [10,11,12] and across various modes of transportation such as pedestrians [13], bicyclists [14], car drivers [15], or public transportation users [16]. However, each of these questions relies on data and their quality determines the quality of the service. Some types of data such as geometry are easy to determine, others such as driving restrictions have to be derived from legal norms, and some such as the tarmac quality can only be determined by visual inspection. The computation of an optimal path or the navigational support requires knowledge on the goal of the navigation. An optimal path for a tourist differs from the optimal path of a commuter even in the case of identical start and end point. Thus, context is relevant in the field of navigation. It can be determined by observing and classifying human behaviour. Tourists move differently than computers, or people on a shopping trip. Even familiarity with the environment has an impact on the behaviour. Alinaghi et al. [17], for example, showed that head movement is an indication of spatial familiarity, which has an impact on suitable routes and information demand [18].

Therefore, merging human behaviour assessment with research of navigation is relevant to improve navigation systems and to adapt them to a larger variety of situations. However, the limitations connected to the area of observation restrict the sensors used in navigation. Vehicle navigation is typically is easier to solve because sensors such as GNSS receivers, Inertial Measurement Units (IMUs), or wheel rotations sensors are already built into the vehicle or can be easily attached. However, most of the systems used in vehicles assess the movement of the vehicle only and ignore the human behaviour. An Anti-lock Braking System (ABS), for example, only releases the break in case of a locked wheel. Other systems have to deal with more complex dynamics. A motorcycle rider airbag uses IMUs to activate the airbag. Early tests used an IMU on both the motorcycle and the motorcyclist [19]. However, this study uses a simplified setup because it is track-based, i.e., the motorcyclists are racers and the situation is race-like. A few years later, wearable motorcyclist airbags were available for road usage as well and they only used sensors in the airbag vest. Still, all these systems have a similar service limitation: They are intended to identify a single situation, where the body of the motorcyclist is in danger of hitting the ground. Assessing the intention and thus the behaviour of the motorcyclist is more complex but it would also provide more information than a simple system such as an ABS. Because the human behaviour is affected by environmental factors information on the road condition, the visibility, or the traffic situation, can be derived from it.

As already stated, behaviour of navigating persons should be potentially monitored on all roads all the time. This also applies for the assessment of motorcyclist behaviour. Cameras will not cover all urban and rural roads. Therefore, sensors that the motorcyclist carries or sensors that are already attached to the motorcycle should be used as data sources. Typical sensors carried by humans include mobile phones. Modern phones support GNSS positioning and are frequently used to navigate. GNSS has, for example, already been used to cluster bend-taking practices of motorcyclists [20]. However, urban canyons or forest areas restrict the quality of positioning and the method provides mainly information on the roads used and the speed of travel. This is sufficient to distinguish between modes of transportation (walking, cycling, and riding a motorcycle) or to identify vehicle stops [21], but analysis beyond that is challenging. Thus, sensors on the motorcycle should be used but not all available sensors are equally well-suited. Shahverdy et al. [22] used driving signals such as acceleration, throttle, speed, and Revolutions Per Minute (RPM) to recognize the state of car drivers (including the categories aggressive, distracted, and drunk). These driving signals might be misleading for motorcycles due to the drive-by-wire approach. Throttle input by the motorcyclist, for example, might be adapted by the engine electronics due to other sensor readings and system settings. Thus, this paper focuses on sensors that observe the movement of the motorcycle itself since they should be less affected by corrections. Modern motorcycles do have an ABS and many of them even have a cornering ABS. The latter one includes the lean angle in the assessment of the adequate response. Other support systems, such as the anti-spin system for the rear wheel or the wheelie control, need additional information, e.g., lateral acceleration or pitch and the height difference between the front and back wheel. Therefore, these motorcycles have a built-in IMU. Maceira et al. [23], for example, use such a sensor to estimate the roll angle of a motorcycle. However, the sensor can also provide more data for behaviour assessment.

Humans adapt their behaviour in response to the environment. Heavy rain, for example, often changes the behaviour of pedestrians. They may walk faster, select other paths, or simply keep more space to other pedestrians because the umbrellas need space. The same is true for motorcyclists because the water on the road poses a potential hazard that they must consider. However, there are more aspects that are relevant, such as road quality, traffic density, motorcyclist training, or trip purpose. This paper analyses if an assessment of the motorcyclist behaviour is possible such that in a follow-up step the data can be used to collect information on current traffic situation and road status. To the best knowledge of the authors, this issue has not been sufficiently addressed by previous works.

The contributions of the paper are manifold. Based on a set of different types of motorcyclist behaviour, the paper covers the following:

- Define a process to collect and analyze IMU data;
- Show the quality of a model created with XGBoost to classify motorcyclist behaviour;
- Use feature importance analysis to identify the most relevant features for each type of behaviour;
- Discuss application scenarios for such a classification.

The corresponding research questions are as follows:

- Are the differences between the various types of riding behaviour significant enough to distinguish between them?
- Is it possible to train XGBoost to classify collected data?
- What accuracy can be achieved?

The advantage of a classification model purely based on an IMU is that once the built-in IMU is accessible, the data can be collected directly at the motorcycle. No additional hardware is required and therefore, the data could be collected by a large number of motorcyclists. However, the design must also consider ethical concerns. It is essential that data are only collected if the motorcyclist consents. In addition, only classified data and no original IMU data should be provided. This has some advantages. In the context of privacy, the classified data contain less information than the original data, which could, for example, allow assessment of the driving speed. In the context of scalability, the amount of data transferred to a central storage is reduced. In addition, the necessary computational power is required on the vehicle and not on the storage facility. The last two aspects are relevant if a large number of motorcyclists contribute data.

This work focuses on the behaviour of the motorcyclist, which is influenced by the motorcyclist himself and external factors such as the traffic situation or road conditions. It does not deal with the road safety of motorcycles. This would include aspects such as speeding [24], visibility [25], and fatigue [26], and these topics have already been addressed in literature. Yousif et al. [27] provide an overview and propose a framework to analyse motorcycle accidents. This is in line with what was achieved 20 years ago [28], and only some classes are missing, e.g., stunts (high-risk manoeuvrers).

The remainder of the article is structured as follows: Section 2 contains the set of assumptions used for the experiment, the setup for data collection, and the procedure for data analysis. Section 3 shows the results obtained. The discussion of these results is documented in Section 4. A summary of the results and a discussion of open questions concludes this work.

## 2. Materials and Methods

### 2.1. Assumptions of the Paper

Similar to cars, modern motorcycles do have a number of different sensors that are attached to them. These sensors not only enable optimal combustion in the engine but also avoid blocking of wheels during braking, ensure that both wheels have contact with the road, or restrict wheel-spin during acceleration. These sensors observe, for example, the rotation speed of both wheels and the acceleration of the motorcycle along the three axes, i.e., modern motorcycles have a built-in IMU. To the authors’ knowledge, there is currently no method to access the data stream of these sensors without losing the warranty for the vehicle. The authors contacted the producer of the motorcycle used for the experiment. Although the producer recorded sensor data in the model development and testing phase, the production motorcycles do not provide this possibility. Therefore, an external IMU was attached to the motorcycle, and the data was recorded on a laptop computer.

### 2.2. Experimental Design and Workflow

The experiment consisted of several steps:

1. In the first step, the first author mounted an IMU and a camera on his motorcycle and drove along a predefined route on 11 different days. IMU data were recorded on a laptop and the camera recorded on an internal memory card. The synchronization between IMU data and camera data was coarsely performed by filming the notebook clock and fine-tuned by a set of motions after mounting IMU and camera on the motorcycle. The motions were sharp rolls of the standing motorcycle to the left and right, which could easily be identified in the video as well as in the IMU data;
2. The video was classified manually according to the selected types of behaviour and the time-codes of the mode changes were written in a text file;
3. The text file with time-codes was used to identify segments in the IMU data that describe a specific behaviour;
4. The segments were split into equal intervals to get IMU sequences with equal information content for the statistical parameter calculation;
5. The interval data were described by a set of statistical parameters (the features), which were then inserted into XGBoost. A split of 70% and 30% was used for training and validation and a 10-fold cross validation was used for the quality assessment;
6. SHAP was applied to identify the most important features.

**Figure 1:**
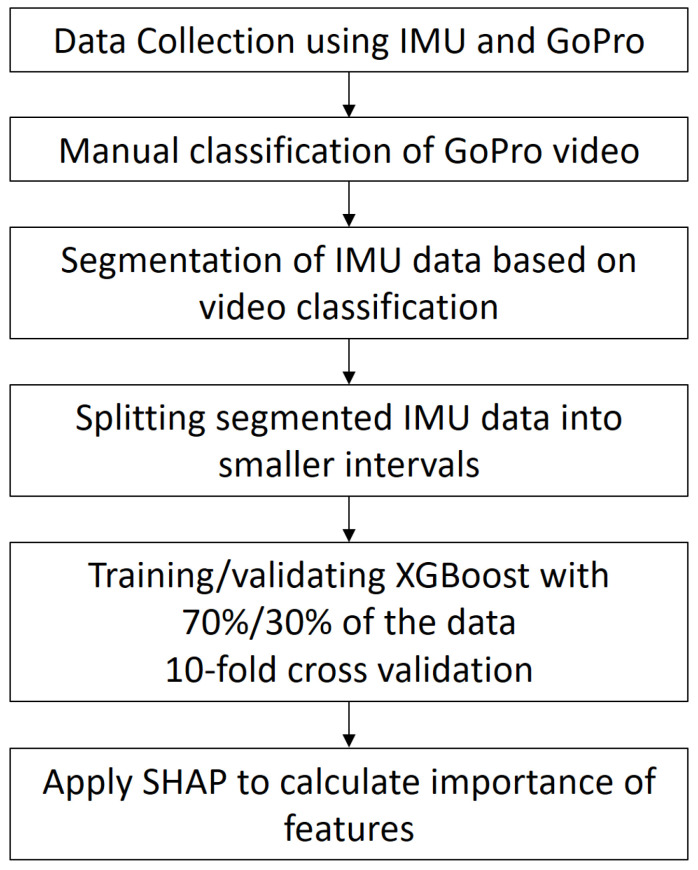
Workflow of the experiment.

Figure 1 shows the different steps graphically.

The goal of this design is to collect data that allows to answer the question concerning the potential of IMU data to identify the different types of behaviour and to provide information on the quality of the evaluation. Variations of the weather condition were minimized by the selection of test days based on the weather condition.

### 2.3. Collection of Training Data Set

Two slightly different motorcycles were used:

- KTM Duke 790 in Summer/Fall 2021;
- KTM Duke 890 in Spring 2022.

The change of motorcycle was necessary due to theft and the Duke 790 (KTM Sportmotorcycle GmbH, Mattighofen, Austria) was not in production at the time of replacement. The technical specifications are almost identical (see Table 1). The main difference is the slightly larger engine, resulting in more power and torque and slightly more weight. The overall setup, however, is almost unchanged and thus the driving behaviour of both motorcycles is identical.

The IMU used for the experiment was an XSENS MTi (Movella Inc, Henderson, NV, USA) [29]. The IMU was attached to the fuel tank cap using hook-and-loop tape. The data from the IMU was recorded on a Microsoft Surface Book 2 (Microsoft Corporation, Redmond, WA, USA) using the software provided with the IMU. The computer was stored in a backpack during the tests.

**Figure 2:**
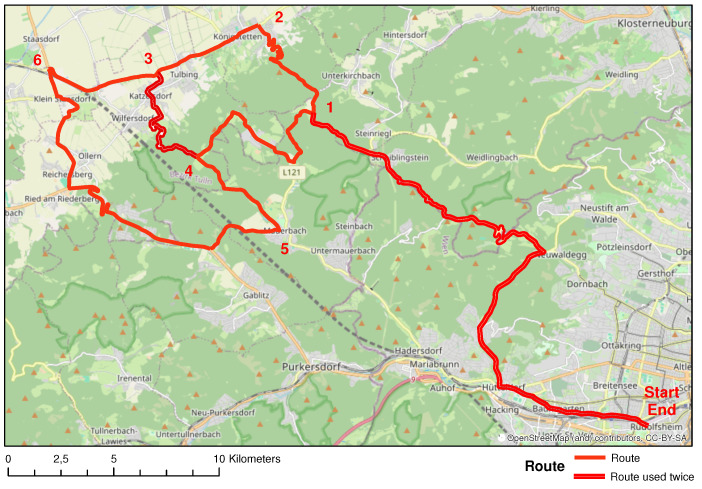
Route of the test rides: Start-1-2-3-4-5-6-3-4-1-End. Background: OpenStreetMap.

The route to collect the data is 93 km long (see Figure 2). The route consists of different types of road: urban, rural primary roads, and rural secondary roads. Table 2 shows the dates when the test rides took place, the motorcycle used, and the person riding the motorcycle. The weather conditions on all days were sunny and the road surface was dry. Temperatures were always above 25 °C.

Only two different motorcyclists of similar experience participated in the experiment and two similar motorcycles were used. In addition, the total distance is approximately 1000 km. The sample is therefore quite small and homogeneous. This might weaken the reliability of the experiment. However, the approach is still realistic because the raw data should be processed on the motorcycle to prevent privacy issues. This would usually prevent that too many different motorcyclists are involved in the classification process.

**Figure 3:**
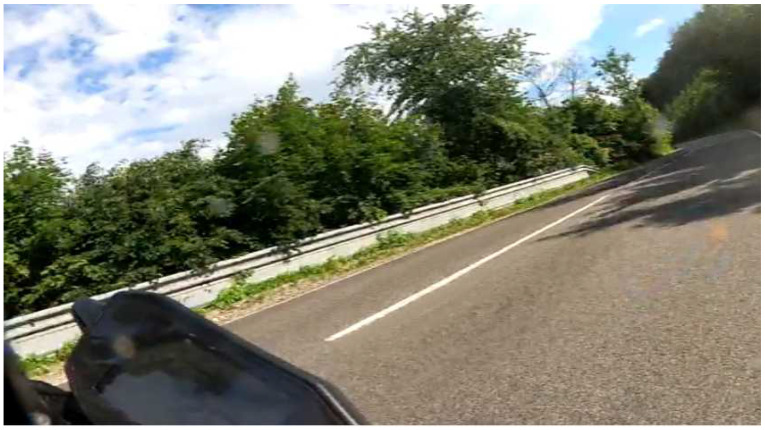
Snapshot from one of the videos.

The data collection process was recorded with a front-mounted GoPro (see Figure 3). These videos allowed for a visual inspection of the various situations along the route and thus a manual classification of the collected data from the motorcyclist.

### 2.4. Data Preparation for Further Analysis

The following attributes were collected with a sample frequency of 50 Hz during the rides:

- PacketCounter;
- SampleTimeFine;
- Acc_X, Acc_Y, Acc_Z: Accelerations along the sensor/motorcycle axes;
- Gyr_X, Gyr_Y, Gyr_Z: Angular accelerations based on the sensor/motorcycle axes;
- Mag_X, Mag_Y, Mag_Z: Magnetic field values;
- VelInc_X, VelInc_Y, VelInc_Z: Incremental accelerations along the senor/motorcycle axes;
- OriInc_q0, OriInc_q1, OriInc_q2, OriInc_q3: Quaternion description of the incremental rotations in the sensor/motorcycle coordinate system;
- Roll, Pitch, Yaw: Euler angles of the sensor/motorcycle coordinate system in the earth-fixed coordinate system;
- Motorcyclist: Name of the person moving the motorcycle.

A problem that can occur in the data is the interference between engine vibrations and sampling frequency. The engine vibrations vary with the Rotations Per Minute (RPM) in the range of approximately 1000 to 6000 RPM. We tried to minimize the effect by loosely mounting the IMU to the motorcycle using a hook-and-loop fastener. Thus, the IMU moved with the motorcycle but had some freedom to dampen vibrations.

The first task of the analysis is that the identification of relevant behaviour needs to be identified. Optimal riding conditions in terms of weather, grip level, and traffic allow motorcyclists to select the driving speed according to their personal preference. This can be rather quick, which requires deceleration before corners and acceleration afterward, or in a more relaxed driving speed with less or no acceleration or deceleration. The latter style may also be relevant when the legal speed limit severely restricts driving speed. The first style is labelled as “fun” in this work, the second one as “cruise”. However, external factors such as the grip level or traffic can restrict the driving speed even further and can affect the breaking behaviour, resulting in slower deceleration than usual. Situations, where the motorcyclist is following cars, might require breaking manoeuvrers in corners or on straight roads, a behaviour that is rather untypical for motorcyclists. This kind of behaviour will occur not only in urban areas but also on rural and winding roads in case of traffic. Similar to other traffic participants, motorcyclists have to stop at red traffic lights or at intersections with stop signs. In the case of a stop sign, a long waiting time indicates dense traffic. Finally, overtaking another vehicle is a frequent behaviour also related to traffic. Our assumption is that different kinds of behaviour lead to different patterns in the IMU data. The identified classes are the following:

- “fun”: This behaviour is characterized by strong acceleration and deceleration, large lean angles, and a high frequency of change in all parameters. Typically, roads have a large number of different bends, and this leads to frequent changes in lean angle and driving speed;
- “cruise”: This behaviour is similar to “fun” but it is smoother. Deceleration, for example, may be achieved by throttling back instead of applying the brakes, resulting in weaker deceleration. Usually, the lean angles are also lower than in the behaviour “fun”.
- “traffic”: This behaviour is heavily affected by other vehicles. Therefore, deceleration and acceleration do not necessarily correlate with corners. Strong deceleration might be necessary on straight road segments or in the middle of a corner and in both cases, this is a behaviour that motorcyclists typically try to avoid;
- “wait”: Waiting for a stop sign or a red traffic sign means that the motorcyclist maintains balance by putting at least one foot on the road. This stabilizes the motorcycle and the lean angle variations will be quite small. Acceleration and deceleration will be close to zero since no significant forward movement occurs;
- “overtake”: Overtakes constitute a series of movements. In right-hand traffic, the start of the overtake requires leaning to the left followed by leaning to the right. Before, during, or after this lateral offset of the driving path, the motorcyclist will accelerate the motorcycle. At the end of the overtake, the lateral offset is undone by leaning to the right followed by leaning to the left. Whether there is a deceleration or not depends on the traffic situation.

Table 3 summarizes these properties. Although there will be some variation within each of the behaviours, the differences between the classes were expected to be distinguishable by a machine learning algorithm.

In order to prepare the raw data, the following statistical parameters were calculated for each of the attributes: mean, median, standard deviation, maximum, and minimum. This results in 96 features (5 different statistical parameters for each of the 19 attributes and the result of the manual classification).

Each of these statistical parameters was calculated for a small slice of the IMU data. The length of the slices was set to 10 s (sliding window and step size). The time was determined based on the driving school rule that overtake manoeuvrers take 10 s. The use of the 10 s also led to

- Enough data in each slice to calculate reasonable estimates for the statistical parameters;
- Equal size of the slices;
- A sufficient number of slices for the training.

In order to avoid mixed slices (e.g., 5 s “wait”, then 5 s “fun”), the data stream was segmented manually using the video recordings. One problem that could not be eliminated was the length of overtake manoeuvrers since they rarely took 10 s or more. Overall, 2150 slices of class “fun”, 3900 slices of class “cruise”, 3550 slices of class “traffic”, 550 slices of class “wait”, and 100 slices of class “overtake” were produced.

### 2.5. Classification

We utilized the powerful gradient boosting technique to build predictive models, which uses the strength of several weak learners (that is, trees) to produce a strong one. Our implementation of this technique, XGBoost [30], is highly efficient and scalable, making it an ideal choice for our purposes. We selected XGBoost due to our own experience and experiences published by colleagues. Rehrl et al. [21], for example, reported that XGBoost and Random Forests showed the best performance during the analysis of track data. A comparison with other approaches was not performed in this study because the focus is testing the applicability of the approach and not the optimization of the machine learning methodology.

The data were split in 70/30 training and validation sets. We fine-tuned our models’ hyperparameters using a randomized search approach with 10-fold cross-validation. The classification of the data was achieved using trained models, with *mlogloss* plotted to assess for under- or overfitting (with negative log-likelihood serving as our scoring function).

To better understand the results of our models, we utilized the Tree SHapley Additive exPlanations (SHAP) method to calculate the importance of each feature [31]. This approach provides a more comprehensive interpretation of the results, allowing for greater insight and understanding of the underlying data. This is the second task of the analysis.

## 3. Results

**Figure 4:**
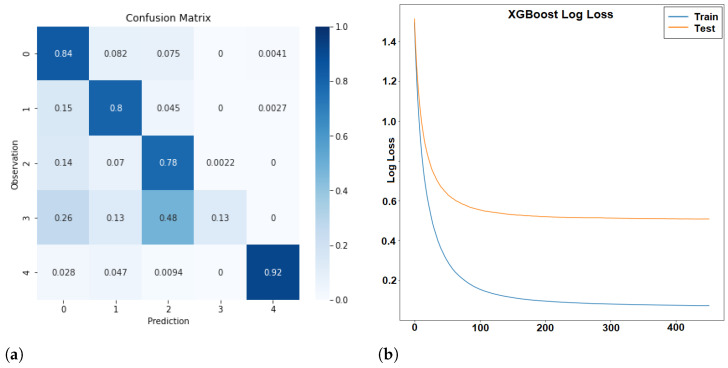
Confusion Matrix (**a**) and Loss (**b**) with negative log-likelihood as a scoring function for all classes: Cruise (0), Traffic (1), Fun (2), Overtake (3), and Wait (4) using all available features.

The first classification attempt resulted in an overall classification accuracy of 80.97%. Figure 4 shows the confusion matrix and loss function of the classification. The loss function (Figure 4b) shows a regular pattern, i.e., there is a slow but regular decrease of the logarithmic loss both for the training and the test data. Thus, no problem of overfitting occurred during training. The confusion matrix (Figure 4a) indicates that some classes are identified with higher accuracy than others. The class “wait” has the highest accuracy with 92%. This might be due to the fact that it is the only class that does not involve movement. The worst class is “overtake”, which is often confused with “fun”. Only 13% of the cases are correctly classified as overtake and 48% as “fun”. The reason might be that an overtake requires a quick change of leaning angles and acceleration, something that is also associated with “fun”. The presumed pattern mentioned in Table 3 was not sufficient to distinguish between the two classes. Another problem might be the different interval lengths of the data in class “fun” and in class “overtake”. Overtake manoeuvrers are usually finished after a few seconds, but the interval for the classification was set to 10 s. Thus, the samples in class “overtake” are shorter than in all other classes, and this might make it even more difficult to identify overtake manoeuvrers.

**Figure 5:**
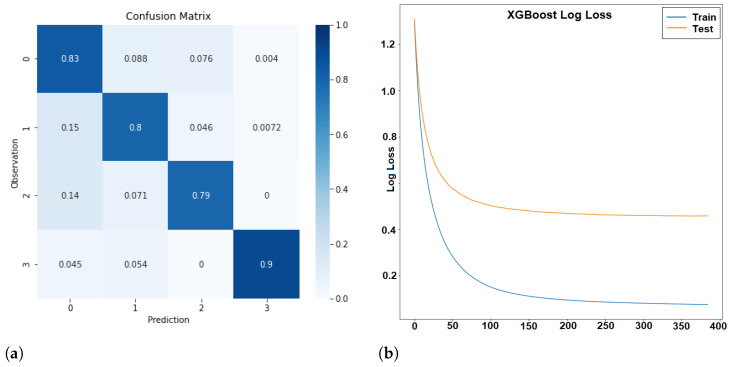
Confusion Matrix (**a**) and Loss (**b**) with negative log-likelihood as a scoring function for the classes Cruise (0), Traffic (1), Fun (2), and Wait (3) using all available features.

In order to eliminate the problem of overtake manoeuvrers from the analysis, a second classification attempt was performed where the class “overtake” was excluded. Figure 5a shows the confusion matrix of the classification. The percentage of correctly classified data for each class is slightly worse than in the previous case except for the class “fun”, which was recognized slightly better than before. However, the overall accuracy increased to 81.56%. Again, the loss function showed a regular behaviour (see Figure 5b).

**Figure 6:**
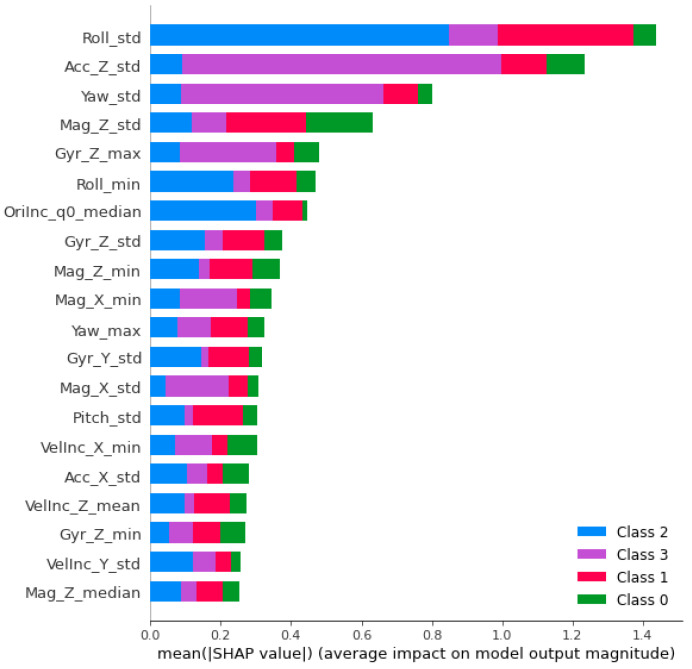
SHAP feature importance.

The SHAP feature importance (Figure 6) shows the effect of the different features in the classification. However, explanations are necessary, which is why the different features are relevant. If no explanation can be found it might just be a pattern inherent in only the training data and thus not representative for the class.

The easiest pattern to explain is class 3 “wait” (the purple bars). The most relevant features are the standard deviation of the vertical acceleration and the standard deviation of yaw. Since the motorcycle is not moving while waiting, it is reasonable that these two values differ from the values for all other features:

- Vertical acceleration: While driving, the vertical acceleration will be influenced by the elevation profile of the road height and by road bumps, e.g., from potholes. Thus, the recorded vertical acceleration contains some variation. This is not the case while standing. The only variation in the vertical acceleration might be a result of driver movement and thus the standard deviation is much smaller during waiting than during driving.
- Yaw: The orientation of the motorcycle (yaw) is determined by the position of the wheels. As long as their position does not change, the orientation does not change either. This is reflected in the IMU data if the sensor is attached to the body of the motorcycle. This was the case in the experiment. The result will be different if the sensor is attached to the front wheel, e.g., as a part of the braking system.

The blue bars represent the weight for assessment of class 2 “fun”. The most important feature is the variation of roll, expressed by the standard deviation. Other important aspects are the minimum roll angle and median q0 component of the quaternion. While the first represents the maximum lean angle, the component of the quaternion is slightly more difficult to explain. The quaternion describes the rotation in space not by the Euler angles (roll, pitch, yaw), but by providing a rotation axis in 3D space and an angle to rotate around this axis. q0 is this angle. Thus, the parameter describes how much the system is rotated over time. The use of the median indicates that the time is relevant when no significant change in the angles occur.

For “traffic” (the red bars) the variation of roll and the vertical component of the magnetic field are most important. However, the variation in pitch is also relevant, reflecting the sharp break manoeuvrers that can occur while driving in dense traffic. However, it is obvious that it requires a lot more different parameters to identify the class “traffic” than to identify the classes “fun” or “wait”.

The class “cruise” (green bars) has no obviously important feature. Some features get higher importance (e.g., Mag_z_std. VelInc_X_min, Acc_X_std, or Gyr_Z_min) than others (e.g., OriInc_q0_median). However, the class seems to be described by the breadth of its features rather than by a single aspect.

## 4. Discussion

### 4.1. Classification Results and Their Improvement

The experiment was initiated under the assumption that motorcyclist’s behaviour can be identified by analysis of data from an IMU. We expected that the differences between the five classes “cruise”, “traffic”, “fun”, “overtake”, and “wait” are large enough to separate them. The results of this work suggest that this is feasible, allowing to utilize such models to reveal the motorcyclists behaviour.

The experiment showed that there are classes that are easy to recognize and classes that might be more difficult due to similarities in the driving behaviour, e.g., overtake and fun exhibit similar behaviour (one could say that overtakes are also fun). Important differences that occur on longer sections such as riding in traffic or having fun were detected with an accuracy of 80%. This already allows specific classifications for trip segments. When adding the position of the vehicle (which is always known through the navigation device), the classification can be attached to a specific road segment or a part thereof. This provides a spatial distribution of the collected classifications.

Obviously, the classification results depend on the road situation. In a country with similar quality of road surface, the hyperparameters might be similar. However, in countries with bumpy tarmac or primarily dirt roads, the hyperparameters might be completely different. However, this does not stipulate a major restriction of the classification since only few motorcyclists do extensive tours that could face dramatic differences. However, there is a simple solution for even this case: Geofencing [32] could be used to stop the classification if the motorcyclist leaves a specific area, e.g., by travelling from one continent to another.

The classification of the overtake manoeuvrers did not work as expected, as only 13% of these manoeuvrers were correctly identified. The problem might be the duration of the event, which usually only takes 2–3 s whereas all other classes were segmented into 10 s intervals. In general, short events might be much more difficult to detect than long-lasting behaviour. This leads to the conclusion that other types of short events might also be difficult to detect, e.g., the avoidance of oil-polluted patches or wet leaves on the tarmac in a curve. A potential method to identify overtakes could be the correlation of roll with the curvature of the road. An overtake manoeuvrer will not follow the changes of curvature. On a straight road, for example, the described pattern will be independent of the road. The same pattern in roll on a street segment that turns left, then right, and then left again would indicate fun and not overtake. This, however, would require detailed knowledge of the road geometry and accurate positioning.

Given enough observations by different motorcyclists at different times will allow to assess, for example, the amount of traffic on a specific road segment or the quality of the road surface. A winding road might be a good choice for riding, but if there is too much traffic, the stress level increases due to the different driving mechanics of cars and motorcycles. Some motorcyclists might be lucky and have no traffic, so their behaviour will be classified as fun. However, most motorcyclists will be blocked by cars that they cannot overtake on the winding road and thus their behaviour will be classified as traffic. Thos is an indication of high traffic density if the second group is much larger than the first one. The classification result might even indicate more than that. If there are ongoing construction works, then traffic lights might stop vehicles temporarily and the speed limit and road condition in the working area will lead to a different classification than suggested by the pure geometry or traffic density information. The same happens for roads with a low-grip surface, e.g., paving stone. However, the availability of positional information is a fundamental prerequisite for merging the classifications collected by different motorcyclists and/or at a different time. This study only used IMU data. The positioning technique must be adopted in a second step to connect the classification result to a specific segment of a road network. The information could for example be delivered by a device containing a GNSS sensor such as a smartphone or a navigation system. Only this step would allow for statistical analysis of the results.

### 4.2. Potential Application Areas

What are the application scenarios for such a classification? The assessed behaviour is connected to locomotion, which is a part of navigation. Thus, the general field of navigation is a realistic application area. Navigation is an essential part of everyday life and many services have been created to support it. Regardless of the implementation, the quality of such a service depends on the quality of the underlying data. Geometrical data are collected by governmental agencies, private parties, or in form of Volunteered Geographic Information (VGI) [33] (for example, OpenStreetMap; www.openstreetmap.org). Additional information includes restrictions such as one-way streets or weight limits and parameters required for the assessment of routing costs. The restrictions are documented by traffic signs, are easy to identify, and are typically valid for extended periods. The parameters required to assess routing costs on the other hand can change rapidly, e.g., the actual driving speed in a narrow road in a city centre (compare Ref. [34]).

Routing applications minimize the costs, typically expressed as travel distance or travel time. However, any other parameter is possible if it can be derived from the attributes of the underlying data. This works well for pedestrians, public transport users, bicyclists, or car drivers. However, when trying to produce reasonable routes for motorcyclists, the available data is insufficient because optimal routes depend on a combination of different parameters such as the following: [35]

- Curviness of the route;
- Inclinations and declination along the route;
- Legal speed limit;
- Landscape around the route;
- Grip level;
- Traffic density, especially trucks.

Only the first two aspects can be derived directly from the geometry of the road and the speed limit is usually part of the additional data.

Traffic density is an important feature also for other user groups. Traffic regulators typically have a general idea on traffic density. Traditionally, they used direct observation, either by human traffic counters or traffic counting infrastructure, or video cameras. The widespread availability of smartphones would theoretically allow the use of these to identify traffic jams but privacy laws typically restrict the methods significantly. Only providers of used operating systems (mainly Google and Apple) have the amount of data necessary to statistically identify traffic density. It is a based on the idea of floating car data, which was already implemented using taxis [36]. A problem with taxis is that they mainly drive in cities. Rural areas are therefore not covered. Motorcycles are used both within cities and in rural areas. Thus, they would provide better spatial coverage. This would help traffic regulators to analyse the traffic situation and work on measures to improve the situation.

Traffic density is also relevant for motorcyclists because the majority of accidents and especially of motorcycle accidents are collisions with other vehicles. The German umbrella organization of insurance companies (Gesamtverband der Deutschen Versicherungswirtschaft e.V., GDV) reports that 76% of the accidents in rural areas are collisions with vehicles such as cars or trucks [37]. They analysed 2345 accidents with motorcycles in Germany before 2020. A total of 85% of the accidents (1988 accidents) included motorcyclists not riding in a group. Additionally, 44% of these accidents (867 accidents) occurred in rural areas. Table 4 shows the statistics and the numbers suggest that riding roads with little traffic creates safety for motorcyclists. However, monitoring traffic density is costly. Jain et al. [38] list three methods to measure traffic density: In situ traffic detector technologies, image or video processing, and finally, vehicular sensor networks (VSN), e.g., probe vehicles (PVs) or floating cars (FCs). The first two require the installation of technical equipment and are therefore only suitable for sensible points. The last can theoretically cover the entire road network, but depends heavily on the distribution of contributing vehicles and only uses the average driving speed to assess traffic density.

Grip level is not available for all kinds of roads because it can change quickly, e.g., due to rain, construction work, falling leaves, dirt patches, etc. However, motorcyclists continuously scan the surface and adapt their riding behaviour, and the differences can be seen in data collected by a motorcycle-mounted IMU [39]. This topic is more relevant for motorcycles than it is for four-wheeled vehicles because a single wheel exceeding the limit of adhesion leads to critical a situation that can result in an accident. Although there are technical systems that can help in such a situation, their potential impact is limited because they can only control the breaking and the propulsion system but have no impact on lateral forces that the friction between wheel and surface has to absorb. Thus, raising awareness of the motorcyclist is an important safety feature.

The beneficiaries of such applications are many-fold. First and foremost, the motorcyclists will benefit because route planning can be improved. When selecting a route, priority can be given to routes where most of the other motorcyclists showed the behaviour “fun” and routes where “traffic” was most common can be circumvented. This increases the safety for motorcyclists and reduces the stress that traffic usually causes. In addition, live updates of collected behaviour along the route currently planned can warn motorcyclists of spots where everybody slowed down significantly, maybe due to an oil spill. Traffic planners and road maintenance organizations can also benefit. Their task is to find problems in the traffic network. Traffic density and waiting times are indicators for planners that a specific part of the network has a capacity problem. In addition, local problems such as the above-mentioned oil spill could be relevant information for maintenance workers because they can concentrate on identified spots with “unusual” motorcyclist behaviour. Finally, motorcycle vendors might also benefit because some companies have already started creating their own smartphone apps that can use the dashboard of the motorcycle for navigational instructions. Better route determination can be an argument for a specific brand.

### 4.3. Open Questions

There is still a lot of work to be done. The sample size is sufficient for machine learning models but not sufficient for more elaborate deep learning approaches. This could be difficult to achieve with the setup used in this work. Since large amounts of data should be collected, an automated method is necessary to collect the IMU data and store them in a central location. The motorcyclist community could contribute to this effort by actively agreeing to provide the data. However, since almost no one owns an external IMU, cooperation of motorcycle manufacturers is necessary to provide access to the data stream of the onboard IMUs. In order to enable checks of the IMU data, videos would be necessary, but presumably, not many motorcyclists would be willing to record and upload videos of their motorcycle trips. A solution to provide limited test capabilities would be to add positional information. Whenever a navigational device is used, this information is available. Thus, the data collection could be done through the navigation device, which obviously needs to be capable of accessing the IMU data stream.

Not only the amount of data needs to be increased, but coverage of more types of motorcycles and different motorcyclists is also necessary. A general model should be able to cover more than just one motorcycle type. It is necessary to investigate whether such a general model can classify with reasonable quality. It might be necessary to train different models based on different motorcycle types and engine specifications because motorcycles such as choppers, touring bikes, or sports bikes might require a different riding style, and this could have an effect on the sensor data. The same is most likely true for the different experiences of motorcyclists. Lean angles, for example, will increase with experience when there is no restriction by dense traffic, and the IMU data should show this. In general, it needs to be tested how much the results of novice motorcyclists or (semi-)professional motorcyclists deviate from the results presented here.

A question to be further analysed is the data segmentation. In this paper, 10 s intervals were used to segment the data. The reason was to avoid a high fragmentation of the results. A road segment where the class “fun” would apply could have straight sections that could be classified as “cruise” if the intervals are too short. A problem of this approach was the identification of overtake manoeuvrers, which are usually much shorter than 10 s. Further experiments are necessary to find a solution for this issue.

The same applies to the output of the IMU data. In this experiment, a data rate of 50 Hz was used. This is most likely too high not only because of the amount of data collected, but also because there might be some engine-related noise in the data. Vibrations from a high-revving engine might have an impact on the data. Variation of the sampling frequency could indicate where the best balance between data size and quality of the behaviour classification lies.

Another issue is the reduction of data channels. The IMU used in the experiment provided a wide variety of values, each in a different channel. Specialized IMUs like those in cornering anti-lock braking systems might not provide this wealth of data. This raises the question, which channels can be eliminated without significant deterioration of the classification quality.

One goal of the experiment was the identification of the motorcyclist’s behaviour. Only some basic classes were used as a starting point. However, it would be useful to identify unusual behaviour. If a number of experienced motorcyclists, for example, avoid lean angles in the same corner, this behaviour might be affected by the condition of the pavement in this specific place, for example, if there is an oil spill. Thus, unusual behaviour with spatial and temporal correlation could indicate specific situations that could then be communicated to other motorcyclists approaching the spot via their navigational device. This could be a feature to improve motorcyclist safety. This could even be relevant for other road users, such as car drivers or bicyclists. However, it needs to be tested first what kind of situations could be identified and how accurate that would be.

Depending on the spatial resolution, even more safety-related applications would be possible. Data could reveal that there is a security risk, for example, spilled oil in a corner or an accident behind a corner. This information could be distributed via the navigation device. However, this kind of service would require many motorcyclists to contribute VGI in form of class data to a web-based platform with as little delay as possible. The integration in a navigational device that can access the motorcycle’s IMU data would be a plausible approach but this requires cooperation by at least the major motorcycle vendors.

## 5. Conclusions and Future Work

The experiment carried out aimed to observe the behaviour of motorcyclists. The results show that some kinds of behaviour or some types of action are quite well identified. The sample size is rather small and does not allow for deep learning approaches; however, it is large enough to provide valuable results with XGBoost, the method chosen for the classification. Although the sample was collected using two different motorcycles, their similar characteristics should result in a coherent sample. The research questions can be answered as follows:

- Are the differences between the various types of riding behaviour significant enough to distinguish between them? They are—partially. Overtake manoeuvrers are often mixed with the class “fun”. A suggested solution for this problem is the combination of IMU data and road geometry;
- Is it possible to train XGBoost to classify collected data? After excluding overtake manoeuvrers the training was successful. The parameters used to identify a class that can be logically explained;
- What accuracy can be achieved? The remaining classes were identified with good quality (80%).

We also presented a list of open questions at the end of the discussion. The used parameters such as interval length, sample rate, and behaviour classes should be analysed. It should also be tested if the concept works with other motorcycles and other motorcyclists as well. In addition, XGBoost might not be the best option for analysis and other approaches should be compared to the results presented here. Finally, merging the IMU data with positional information opens a whole new set of opportunities for analysis.

## Figures and Tables

**Table 1:** Technical specifications of the two motorcycles used for the experiment.

	Duke 790	Duke 890
Power	105 BHP	115 BHP
Torque	86 n m	92 n m
Dry Weight	169 kg	175 kg
Wheelbase	1475 mm	1482 mm

**Table 2:** Data collection information.

Date	Motorcycle	Motorcyclist
3 May 2021	KTM Duke 790	Motorcyclist 1
16 May 2021	KTM Duke 790	Motorcyclist 1
4 June 2021	KTM Duke 790	Motorcyclist 1
6 June 2021	KTM Duke 790	Motorcyclist 1
15 July 2021	KTM Duke 790	Motorcyclist 1
20 July 2021	KTM Duke 790	Motorcyclist 1
19 May 2022	KTM Duke 890	Motorcyclist 1
20 May 2022	KTM Duke 890	Motorcyclist 1
4 June 2022	KTM Duke 890	Motorcyclist 1
5 June 2022	KTM Duke 890	Motorcyclist 2
6 June 2022	KTM Duke 890	Motorcyclist 1

**Table 3:** Summary of sensor readings expected for different riding modes.

Mode	Acceleration/Deceleration	Lean Angle	Lean Angle Changes
fun	strong	high	frequent
cruise	medium	medium	frequent
traffic	soft to strong	low to medium	medium
wait	almost zero	almost zero	almost none
overtake	medium to strong	small	specific pattern

**Table 4:** Accidents and the type of collision associated with them.

Collision with	Number of Accidents	Relative Number of Accidents
Motorcycle	49	6%
Car or Truck	660	76%
Pedestrian or Cyclist	60	7%
Other	4	0%
None	94	11%
Sum	867	100%

## Data Availability

The categorized IMU data can be found in the data repository of TU Wien: https://researchdata.tuwien.ac.at/records/re6xk-ydq75 (accessed on 20 October 2023). We refrained from uploading the original videos used to classify the raw data due to privacy concerns.
